# Altering Brain Amyloidosis by Intra-Lingual and Extra-Nasal Exposure of Aβ Aggregates

**DOI:** 10.3390/cells11213442

**Published:** 2022-10-31

**Authors:** Nazaret Gamez, Javiera Bravo-Alegria, Yumeng Huang, Nelson Perez-Urrutia, Deepa Dongarwar, Claudio Soto, Rodrigo Morales

**Affiliations:** 1 Department of Neurology, The University of Texas Health Science Center at Houston, 6431 Fannin St., Houston, TX 77030, USA; 2 Dpto. Biología Celular, Genética y Fisiología, Instituto de Investigación Biomédica de Málaga-IBIMA, Facultad de Ciencias, Universidad of Malaga, 29010 Malaga, Spain; 3 Universidad de los Andes, Facultad de Medicina, Av. San Carlos de Apoquindo 2200, Las Condes, Santiago 7620001, Chile; 4 Facultad de Ciencias de la Salud, Universidad San Sebastian, Lientur 1456, Concepcion 4080871, Chile; 5 Centro Integrativo de Biologia y Quimica Aplicada (CIBQA), Universidad Bernardo O’Higgins, Santiago 8370993, Chile

**Keywords:** amyloid-beta, Alzheimer’s disease, prion, tongue, nasal cavity

## Abstract

Extensive experimental and human-derived evidence suggest that misfolded Aβ particles spread similarly to infectious prions. Moreover, peripheral administration of Aβ seeds accelerates brain amyloidosis in both susceptible experimental animals and humans. The mechanisms and elements governing the transport of misfolded Aβ from the periphery to the brain are not fully understood, although circulation and retrograde axonal transport have been proposed. Here, we demonstrate that injection of Aβ seeds in the tongue, a highly innervated organ, substantially accelerates the appearance of plaques in Tg2576 mice. In addition, the extra-nasal exposure of Aβ aggregates increased amyloid pathology in the olfactory bulb. Our results show that exposing highly innervated tissues to Aβ seeds accelerates AD-like pathological features, and suggest that Aβ seeds can be transported from peripheral compartments to the brain by retrograde axonal transport. Research in this direction may be relevant on different fronts, including disease mechanisms, diagnosis, and risk-evaluation of potential iatrogenic transmission of Aβ misfolding.

## 1. Introduction

Alzheimer’s disease (AD) is a proteinopathy characterized by the abnormal accumulation of misfolded proteins in the brain [1,2]. These proteins include amyloid-beta (Aβ), in the form of extracellular amyloid plaques, tau, as intracellular neurofibrillary tangles, and α-synuclein in some cases [1,2,3]. Although the etiology of AD is still unknown, the amyloid cascade hypothesis states that Aβ aggregates trigger a cascade of events leading to tau pathology, neuroinflammation, synaptic loss, neurodegeneration, and cognitive decline [4,5,6]. This hypothesis is supported by the fact that familial AD cases are associated with mutations in the Aβ precursor protein (APP) or other proteins associated with its proteolytic processing [5]. Additional evidence is found in experimental systems, including the cerebral amyloidosis and AD neuropathology induced in transgenic animal models overexpressing human Aβ [7,8,9]. Moreover, compelling evidence demonstrate that pathological features in these mice can be accelerated and exacerbated by the exogenous administration of Aβ seeds [10,11,12,13]. The latter suggests the self-propagating properties of misfolded Aβ particles, akin to infectious prions [14].

Prion diseases are a group of transmissible and invariably fatal neurological disorders in which the host prion protein (PrP^C^) adopts a misfolded, β-sheet rich, and aggregation-prone conformation [15,16]. These structurally altered and infectious prion proteins (PrP^Sc^) have the ability to template their misfolded conformations and induce the aggregation of natively folded proteins in a process known as “seeding”, thus further transferring their disease-associated properties [17]. Importantly, certain features of PrP^Sc^ have also been associated with other proteins, including but not limited to Aβ and tau [18,19]. Although AD has extensively been recognized as a non-transmissible disease [20], the prion-like behavior of Aβ as well as its potential role in propagating the disease-causing pathology warrant further investigation. Interestingly, studies have reported human-to-human transmission of Aβ amyloidosis following iatrogenic exposure, highlighting the potential issue of interindividual transmission of AD neuropathology [21,22,23]. Nevertheless, evidence of inter-individual transmission of Aβ misfolding in humans is controversial and, as such, the possibility of human-to-human AD transmission is still speculative [24]. Therefore, the induction of cerebral amyloidosis by exogenous Aβ aggregates and their potential seeding mechanisms should be carefully investigated. Moreover, whether the exogenous administration of Aβ seeds via different peripheral routes presents distinguished efficacy in exacerbating brain amyloidosis remains contentious.

We have previously demonstrated that exogenous administration of Aβ-laden brain homogenates (BH) via intra-cerebral inoculation, as well as other peripheral routes, including intraperitoneal, intramuscular, and ocular, can accelerate cerebral amyloidosis in Tg2576 mice [12]. Interestingly, oral gavages of highly concentrated Aβ-containing BH from mouse and human origin did not significantly exacerbate brain amyloidosis in challenged mice [12]. Based on prion studies, the injection of prions into the tongue is reported as 100,000-fold more efficient than oral ingestion in hamsters [25]. Moreover, Kincaid and Bartz found that the nasal cavity is another effective route for prion infection in hamsters [26]. Therefore, in the present study, we evaluated whether the administration of Aβ-seeds containing BH via intra-lingual injections accelerates pathological changes in the brain of Tg2576 animals. Here, we also explored the role of extra-nasal exposure of Aβ seeds-containing BH in inducing cerebral amyloidosis in Tg2576 mice.

## 2. Methods

### 2.1. Mouse Model

In this study, we used Tg2576 mice as model for AD-like brain amyloidosis (~40% males, *n* = 4–6 animals per group) [9]. These animals overexpress a mutant form of APP with the Swedish familial mutation, developing parenchymal Aβ plaques starting at 8–10 months of age [9]. 50-day-old Tg2576 mice were subjected to extra-nasal, intra-lingual, and intra-cerebral (positive control) administration of Aβ-laden brain homogenates. Non-injected Tg2576 mice were utilized as negative controls. All animals were housed in groups of up to five in individually ventilated cages under standard conditions (22 °C, 12 h light/dark cycle) receiving food and water *ad libitum*. Experimental procedures were conducted in accordance with the regulations of the Center of Laboratory Animal Medicine and Care (CLAMC) and the Animal Welfare Committee (AWC) of the University of Texas Health Science Center at Houston.

At the experimental endpoint (300 day-old), animals were sacrificed by CO_2_ inhalation. Untreated animals at this age display few and small amyloid aggregates that are scattered across the brain and visualized only at high magnifications [10,12,27]. Brains were removed for further analyses. The right hemisphere was immersed in 10% formalin for histological assessments, while the left hemisphere was snap frozen and stored at −80°C for biochemical studies.

### 2.2. Inoculum

A brain homogenate (BH) (10% *w*/*v*) prepared using tissues from aged Tg2576 mice (~20 months old) was used as inoculum. To prepare the brain homogenate, frozen brains were homogenized in PBS supplemented with a cocktail of protease inhibitors (PI, Roche Diagnostics GmbH, Mannheim, Germany) and used for intra-lingual, extra-nasal and intra-cerebral administrations. The resulting samples were pooled, snap-frozen, and stored at −80 °C until use.

### 2.3. Intra-Cerebral Injections

As positive control of Aβ seeding, 50-day-old Tg2576 mice (*n* = 5 animals) were bilaterally injected with 10µL of a 10% Tg2576 BH into the hippocampus (10 µL per hemisphere, AP, −1.8mm; LM, ± 1.8mm; DV, −1.8mm). Animals were anesthetized with isoflurane and stereotaxic injections were performed with a Hamilton syringe (26s gauge, 701N, Hamilton, NV, USA). The site of the incision was sutured and the animals were monitored until recovery.

### 2.4. Intra-Lingual Injections

For intra-lingual injections, 10 µL of the 10% Tg2576 BH were injected in the tongue of isoflurane narcotized 50-day-old Tg2576 mice (*n* = 5 animals) using a Hamilton syringe (26s gauge, 701N, Hamilton, NV, USA). The insoluble levels of Aβ_42_ in the BH were assessed by ELISA to estimate the amount of Aβ_42_ that the animals received after a single intra-lingual administration. Mice received a total of 10 µL of 10% Tg2576 BH (containing 4.7 ng of Aβ_42_) via a single intra-lingual administration.

### 2.5. Extra-Nasal Administration

Extra-nasal administrations of 10% Tg2576 BH were performed in isoflurane-anesthetized 50-day-old Tg2576 mice (*n* = 5 animals). The animals were held upright and 5 µL of the inoculum were administered onto the nostrils while inhalation was monitored. Mice received a total of 10 µL of 10% Tg2576 BH (containing 4.7 ng of Aβ_42_) via a single extra-nasal administration.

### 2.6. Untreated Animals

As negative controls, untreated age-matched Tg2576 (*n* = 4 animals) were utilized to monitor Aβ cerebral accumulation. The injection of Aβ depleted extracts were not used as controls in this study as these samples have been extensively shown to lack of seeding activity [13,27].

### 2.7. Aβ Immunofluorescence and Thioflavin-S (ThS) Staining

Right hemispheres (fixed in 10% formalin) were processed for paraffin embedding. Serial sagittal brain slices of 10µm of thickness were sectioned using a microtome. For Aβ staining, brain sections were deparaffined and treated with 85% formic acid for 5 min at room temperature. Then, sections were incubated overnight with the anti-Aβ 4G8 antibody (specific for human Aβ, Biolegend, 800708, San Diego, CA, USA) diluted 1:1000 in PBS/0.02% *v*/*v* Triton X-100 at room temperature. Sections were washed and incubated with goat anti-mouse secondary antibody, Alexa Fluor™ 594 (Thermo Fisher Scientific, Waltham, MA, USA), diluted 1:500 in PBS, for 90 min at room temperature. Stained sections were washed and cover-slipped with ProLong™ Gold Antifade Mountant (P36930, Thermo Fisher Scientific, Waltham, MA, USA). For ThS staining, sections were deparaffined and incubated with 0.1% ThS (Sigma, St. Louis, MO, USA) diluted in 50% ethanol for 10 min. ThS-stained sections were washed two times in 50% ethanol followed by two washes in PBS. Sections were dehydrated in graded ethanol, cleared in xylene, and cover-slipped with DPX mounting medium (Innogenex, San Ramon, CA, USA).

### 2.8. Image Analysis

For Aβ burden quantification, five stained brain sections (one every 10 µm) were examined and scanned with a 10X objective using a DMi8 microscope (K5 microscope camera, Leica, Buffalo Grove, IL, USA). Insets pictures were photographed with a 20X objective. Cortical and hippocampal 4G8 burden as well as ThS-positive amyloid deposits were quantified using the ImageJ software 5.0 (National Institutes of Health, Bethesda MD, USA). 4G8- and ThS-burden were expressed as the percentage of the area stained by the 4G8 antibody or ThS staining versus the total area analyzed (cortex or hippocampus).

### 2.9. Attack Rate

To determine the attack rate, the 4G8 burden in cortex and hippocampus was utilized (Mean ± SD). The attack limit was set at three standard deviations (3σ) over the mean of untreated mice as previously described [12]. Individual values above the attack rate threshold were defined as positive for Aβ seeding induction. Fold increase was calculated using experimental 4G8 burden versus that observed in untreated animals.

### 2.10. Insoluble Aβ_42_ Quantification by ELISA

Left frozen hemispheres from experimental animals were homogenized at 10% *w*/*v* in PBS supplemented with PI, as described previously. BHs (200 µL of 10% *w*/*v*) were ultracentrifuged at 32,600 rpm for 1 h at 4 °C using a 42.2Ti rotor (Beckman-Coulter, Brea, CA, USA). Pellets were resuspended in 200 µL of 70% formic acid following sonication. Then, samples were ultracentrifuged for 30 min at 32,600 rpm (4 °C) and supernatants were collected. To neutralize the acidic pH, formic acid fractions were diluted in 1M Tris buffer pH 11 (1:20) (Sigma-Aldrich, Saint Louis, MO, USA). Aβ_42_ concentration of the formic acid soluble fraction (PBS-insoluble fraction) was analyzed and biochemically quantified using a human-specific Aβ_42_ ELISA kit (Invitrogen, Waltham, CA, USA). The ELISA assay was performed following the manufacturer’s recommendations and absorbance was measured at 450nm using the plate reader EL800 (BioTek, Winooski, VT, USA).

### 2.11. Statistical Analysis

The normal distribution of the data was analyzed by using Kolmogorov–Smirnov or Skewness-Kurtosis statistic tests. We compared the attack rates and 4G8 deposit rate in the olfactory bulb in the extra-nasal and intra-lingual groups using Fisher’s exact test. Next, independent samples t-tests were used to compare 4G8 burden among each of the experimental groups in comparison to the control group. One-way analysis of variance (ANOVA) followed by a multiple comparison test (Tukey) was used to analyze statistical differences among the experimental and control groups. The values are expressed as mean ± standard deviation (SD). Data was analyzed using the Graph Pad Prism software version 9.0 (GraphPad, San Diego, CA, USA). Statistical differences were considered significant for values of *p* < 0.05 (* *p* < 0.05, ** *p* < 0.001).

## 3. Results

### 3.1. Acceleration of Brain Aβ Deposition after Intra-lingual Administration of an Aβ-Laden Brain Extract

First, we validated the Aβ seeding activity of the inoculum used in this study. For that purpose, 50-day old Tg2576 animals were intra-cerebrally challenged with the brain extract described in Methods. Treated animals were sacrificed at the age of 300-day-old and their brains were examined for 4G8 (total Aβ) staining. As shown in Supplemental Figure S1, i.c. inoculated Tg2576 mice developed a massive amount of 4G8-positive Aβ deposits in the cortex and hippocampus compared to untreated animals (Supplemental Figure S1A–E), thus validating the Aβ seeding capacity of the inoculum utilized in this study.

**Figure 1:**
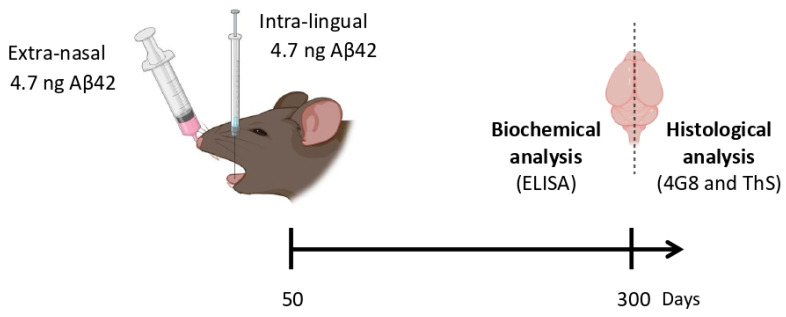
Experimental strategy. 50-day-old Tg2576 animals were challenged with old Tg2576-brain homogenate by two different routes, including extra-nasal or intra-lingual. For extra-nasal administration, animals received 5 µL of BH onto each nostril. For intra-lingual injections, 10 µL of BH was injected into the tongue. The levels of insoluble Aβ_42_ present in the BH were analyzed by ELISA. The estimated amount of administered Aβ_42_ was 4.7 ng by each route. Animals were sacrificed at the age of 300-day-old, brains were removed, and histological as well as biochemical analyses were performed.

**Figure 2:**
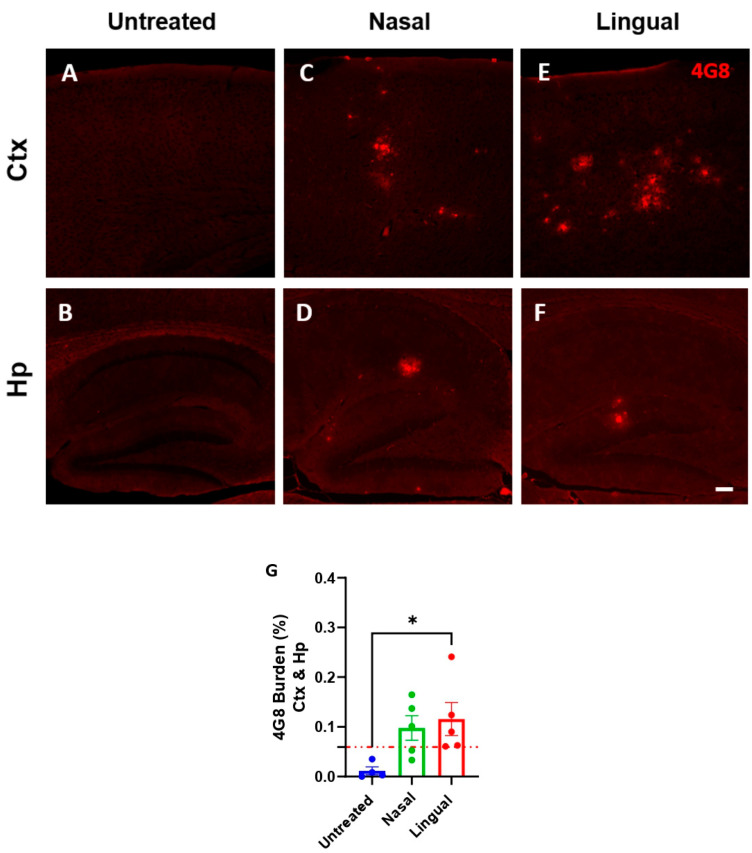
Acceleration of Aβ deposition in Tg2576 mice after exogenous administration of Aβ seeds by an intra-lingual injection. Representative pictures of 4G8 (total Aβ) staining in cortex (**A**–**E**) and hippocampus (**B**–**F**) of untreated, extra-nasally or intra-lingually treated animals. 4G8 burden (%) quantification in cortex and hippocampus (**G**) of experimental animals. 4G8 burden is expressed as the percentage of the area stained by the 4G8 antibody versus the total area analyzed. Attack rate is represented as punctuated red lines in the graph. Values are expressed as mean ± SD. Statistical analyses were performed using One-way ANOVA and Tukey test (* *p* < 0.05). Scale bar represents 100 µm.

After the validation, 50-day old Tg2576 mice were challenged with the Aβ-laden brain extract by two different routes, including intra-lingual and extra-nasal (Figure 1). The estimated amount of Aβ_42_ that animals received in a single intra-lingual or extra-nasal administration was 4.7 ng. Animals were sacrificed at 300 days of age (250 days post-injection) and their brains were removed. To analyze brain Aβ amyloidopathy, the right hemisphere was formalin-fixed and used for histological analyses (4G8 and ThS staining) while the left hemisphere was snap freeze and utilized for the biochemical studies (ELISA). Untreated age-matched animals were used as negative controls for this study, as they show little to no Aβ deposits at 300 days of age (Figure 2A,B).

While several brain regions are susceptible to amyloid seeding in the Tg2576 model, mice in this study were mostly afflicted in the cortex and the hippocampus. Along this line, Aβ amyloidopathy was initially assessed by immunofluorescence in these two brain regions (Figure 2). As observed in Figure 2C,D, extra-nasal inoculated animals displayed a relevant amount of Aβ deposition in the cortex and hippocampus, albeit not significant to that observed in untreated mice (Figure 2G). Interestingly, intra-lingual challenged animals depicted significantly higher levels of 4G8-positive Aβ deposits in the cortex (Figure 2E) and hippocampus (Figure 2F), compared to untreated negative controls (Figure 2G). In agreement with previous prion studies [25], these results suggest that intra-lingual administration of Aβ-containing BH induces exogenous amyloid seeding in predisposing animals. When analyzed in more detail, we observed that changes were mostly restricted to the cortex (Supplemental Figure S2). Interestingly, this pattern resembles the profile observed in aged Tg2576 mice or young Tg2576 mice exposed to Aβ seeds through eye drops, but not to equivalent animals receiving misfolded proteins by intra-cerebral administrations [12].

Moreover, based on the cortical and hippocampal levels of Aβ, we observed that the extra-nasal treated group reflected an incomplete attack rate (3/5), meaning that only 3 out of 5 extra-nasal challenged animals displayed a 4G8 burden above the limit threshold (Table 1). The limit threshold was established at three standard deviations (3σ) over the hippocampal and cortical 4G8 burden mean of the untreated control group (0.01163 ± 0.04794). Notably, the intra-lingual treated group displayed a complete attack rate (5/5), thus further confirming the efficacy of this route to induce exogenous brain amyloid seeding. Fisher’s exact test demonstrated a statistically non-significant difference between the extra-nasal and intra-lingual attack rate (*p* = 0.44). We further observed a statistically significant difference in the 4G8 burden values in the intra-lingual group in comparison to the control group (*p* = 0.03).

**Figure 3:**
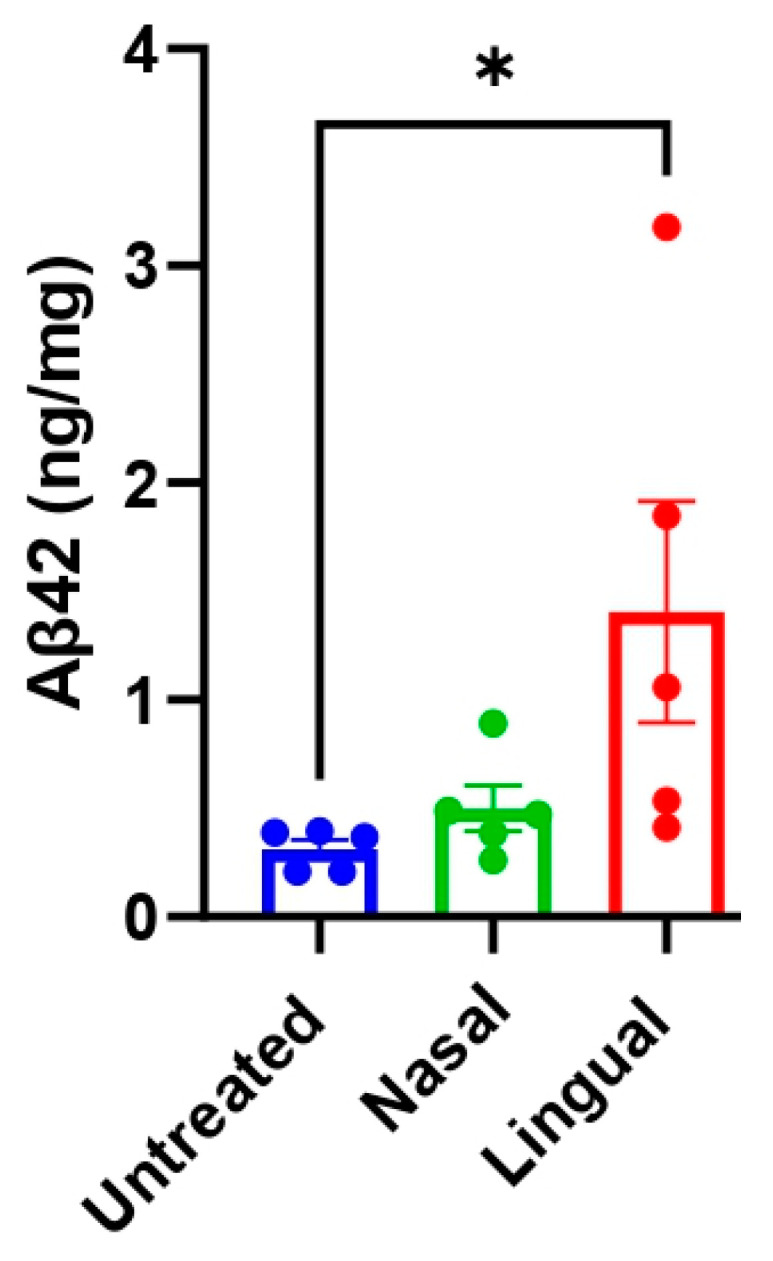
Assessment of brain PBS-insoluble Aβ_42_ levels in experimental Tg2576 mice by ELISA. The formic acid-soluble fraction was biochemically assessed to determine the insoluble Aβ_42_ concentration in experimental and untreated animals as explained in Materials and Methods. Values are expressed as mean ± SD. Statistical analyses were performed using One-way ANOVA and Tukey test (* *p* < 0.05).

Biochemical analyses by ELISA are well-acknowledged to be more sensitive than histological techniques. For that reason, we measured the concentration of PBS-insoluble Aβ_42_ in the frozen left hemisphere of the same mice analyzed in Figure 2 through this method. Thus, ELISA analysis was not restricted to the cortical and hippocampal areas, but instead examined the insoluble levels of Aβ_42_ in the whole brain hemisphere. ELISA results mostly corroborated the histological studies, as the intra-lingual treated group, but not the extra-nasal, displayed significantly higher levels of insoluble Aβ_42_ in the brain of experimental animals compared to untreated control mice (Figure 3). Therefore, these results suggest, once again, the efficacy of the intra-lingual route to exacerbate brain amyloidosis by the administration of exogenous Aβ seeds.

### 3.2. Abundant Deposition of Cerebral Nasal-Seeded Aβ in the Olfactory Bulb of Treated Mice

**Figure 4:**
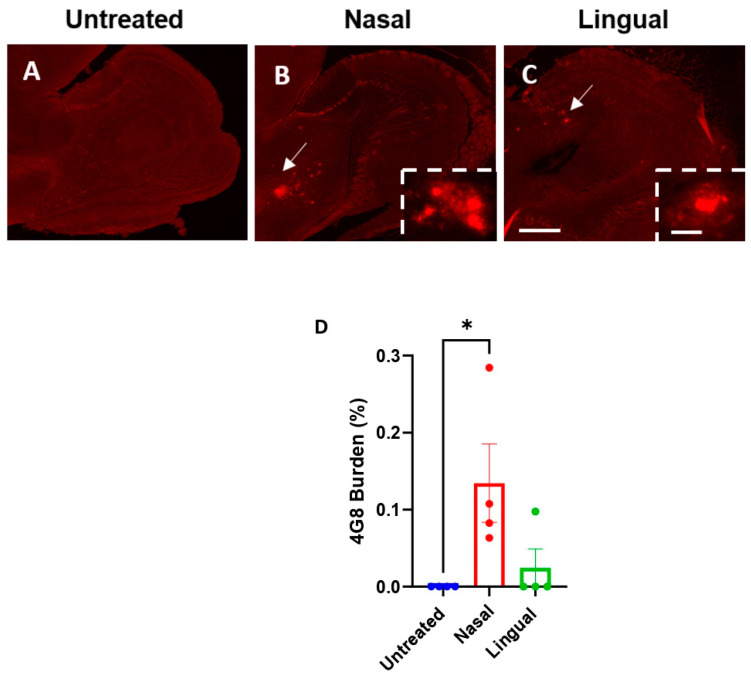
Aβ staining in the olfactory bulb of untreated and misfolded Aβ-treated Tg2576 animals. Representative pictures of 4G8 immunofluorescence in the olfactory bulb of untreated animals (**A**) and mice treated with Aβ-laden BH via extra-nasal or intra-lingual administration, White arrows denote amyloid deposits (**B**,**C**). 4G8 burden (%) quantification in the olfactory bulb (**D**) was statistically analyzed using One-way ANOVA and Tukey test (* *p* < 0.05). Values are expressed as mean ± SD. Scale bar represents 100 µm and 50 µm (insets). White arrows denote amyloid deposits.

Compelling evidence demonstrate that the first areas targeted by prions after peripheral administration depend on the administration route [28]. Interestingly, we previously showed that the prion-like transmission of Aβ misfolding induced the deposition of amyloid plaques in different brain regions in a route of administration dependent fashion [12]. In this context, we examined in detail the presence of 4G8-positive Aβ deposits in brain regions other than the cortex and hippocampus to assess for possible differential tropisms induced by the extra-nasal and intra-lingual administration of Aβ seeds. Notably, we observed that all mice in the extra-nasal group developed 4G8 deposits in the olfactory bulb (Figure 4B). Consistently, the 4G8 burden in this brain region was significantly higher compared to the untreated controls (Figure 4D). Remarkably, only 2/5 intra-lingual treated mice displayed 4G8 deposits in the olfactory bulb, whereas all of the 5 extra-nasal treated mice displayed 4G8 deposits (*p* = 0.17). We also observed a lower 4G8 burden compared to the extra-nasal group, although this difference was not statistically significant (*p* = 0.09) (Figure 4C,D). Four intra-lingual induced animals are represented in the graph in Figure 4D, as data from the fifth was a significant outlier (*p* < 0.05), and thus removed from the analysis. Moreover, quantification of the 4G8 burden in the olfactory bulb showed statistically significant differences between extra-nasal and untreated groups; and non-significant differences between intra-lingual and untreated groups (Figure 4D).

### 3.3. Nasal- and Lingual-Administered Seeds Induce Fibrillar Aβ Deposits in the Brain of Injected Animals

**Figure 5:**
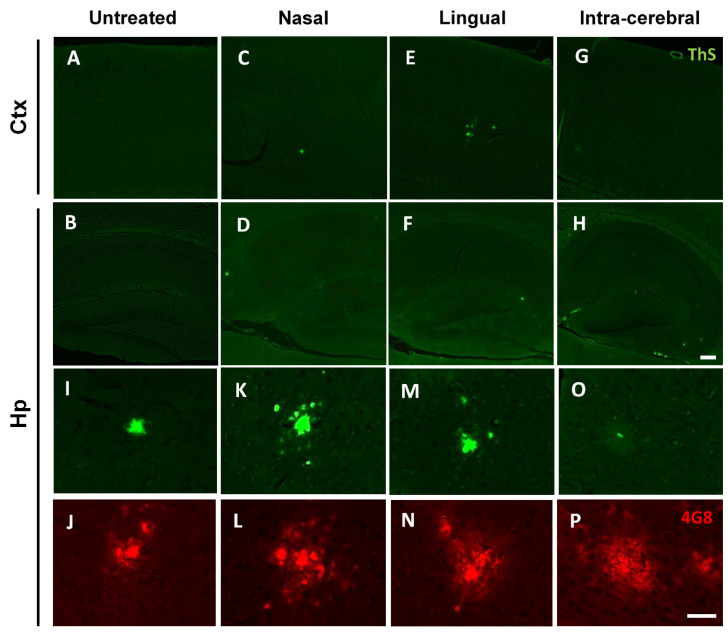

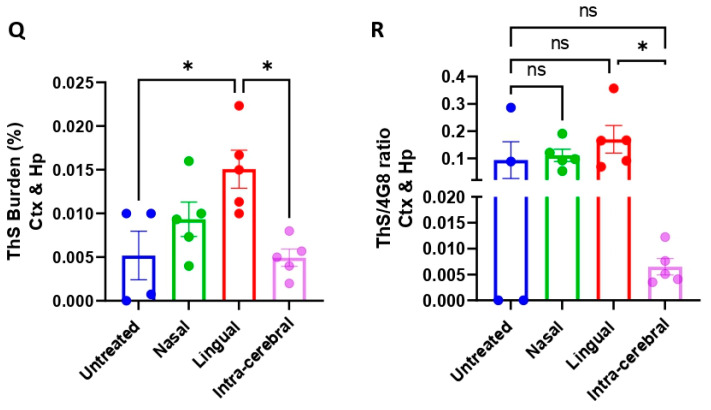
Thioflavin-S staining in the cortex and hippocampus of experimental and control animals. Representative pictures of ThS staining for analysis of fibrillar Aβ aggregates in the cortex (**A**–**G**) and hippocampus (**B**–**H**) of control and experimental mice. Higher magnification pictures of Aβ plaques in the hippocampus after staining using the ThS dye (green, **I**,**K**,**M**,**O**) or the 4G8 antibody (red, **J**,**L**,**N**,**P**) in the consecutive brain section. ThS burden (%) quantification in both cortex and hippocampus brain areas of untreated and extra-nasal or intra-lingual challenged animals (**Q**). Individual values in O were divided by the 4G8 burden found for each animal group as displayed in Figure 2G to calculate the ThS/4G8 burden ratio (**R**). Values are expressed as mean ± SD. Statistical analyses were performed using One-way ANOVA and Tukey test (* *p* < 0.05). Scale bar represents 100 µm (**A**–**H**) and 50 µm (**I**–**N**). ns: not significant.

To further elucidate the properties of the seeded Aβ aggregates in the brain of extra-nasal and intra-lingual infused animals, Thioflavin-S (ThS) staining and quantification analyses were performed. ThS is a fluorescent dye with affinity for fibrillar and highly compact Aβ structures. Notably, some cortical and hippocampal amyloid deposits of either the extra-nasal or intra-lingual groups showed to be positive for ThS staining (Figure 5C–F). We observed that the Aβ aggregates of animals intra-cerebrally injected with old Tg2576 BH were mainly diffuse and displayed poor reactivity against ThS (Figure 5G,H). This interesting feature, that has been previously described by us [12], is reminiscent to what is observed in the brains of patients exposed to pre-formed Aβ seeds [24] and is currently being investigated in our laboratory. Importantly, higher magnification images from the experimental animals in this study demonstrated that both extra-nasal and intra-lingual infused animals likely depicted 4G8-positive Aβ deposits with a compact ThS-positive core surrounded by diffuse Aβ aggregates (Figure 5I,K,M,N). On the contrary, 4G8 deposits of intra-cerebrally injected animals were mostly negative for ThS staining (Figure 5O,P). Quantification of ThS burden in the cortex and hippocampus showed a significant increase of fibrillar amyloid deposits in the brain of intra-lingual injected animals when compared to the untreated group (Figure 5Q). As the cortical and hippocampal 4G8 burden of intra-lingual injected animals were also shown to be significantly higher compared to negative controls, we normalized ThS burden for the 4G8 burden analyzed in Figure 2. As expected, we observed that ThS/4G8 burden ratio was not significantly different among the untreated, the extra-nasal, and intra-lingual groups (Figure 5R), suggesting that the increase of ThS-positive amyloid deposits in the intra-lingual group reflected the relatively higher levels of 4G8-positive aggregates.

## 4. Discussion

Emerging evidence derived from humans and experimental models demonstrate an active cross-talk between the central nervous system (CNS), blood and peripheral tissues in the context of AD [12,29,30,31,32,33,34]. Although more prevalent in the brain, Aβ deposits have been identified in peripheral tissues such as skin, liver, aorta, and others [35]. The importance of peripheral Aβ pools in AD is further demonstrated by the fact that circulating monomeric Aβ contribute to brain amyloid plaques [36,37,38], and that damage in peripheral tissues associated with Aβ clearance enhance pathological progression [30,34,39,40,41]. Moreover, several reports suggest that disease-associated Aβ particles circulate in biological fluids such as the cerebral-spinal fluid and blood [42,43,44,45]. Considering the above-mentioned evidence and the well-established prion-like properties of misfolded Aβ [18], it is plausible that altering the equilibrium of this disease-associated protein in the periphery may have important implications in the progression of AD. Along this line, it has been shown that the intra-muscular administration of cadaveric human growth hormone preparations containing misfolded Aβ particles can induce brain amyloidosis in humans in a manner akin to the iatrogenic transmission of Creutzfeldt-Jakob disease [22,46]. This was further confirmed in experimental settings by inoculating susceptible mice with Aβ-laden brain extracts in their muscles [12]. Importantly, other Aβ administration routes, such as intra-peritoneal, intra-venous, and ocular exposures also promote pathological changes in the brain [12,27,47]. At present, the mechanisms governing the transport of Aβ seeds from peripheral compartments to the brain are still elusive.

Previous studies demonstrate that upon peripheral administration, Aβ seeds are taken by macrophages that could help in their spread [29]. The role of circulatory components in the transport of Aβ seeds to the brain find support in the fact that peripherally treated mice display amyloid deposits associated with blood and meningeal vessels [12]. Nevertheless, other mechanisms, such as retrograde axonal transport, cannot be discarded considering the strong evidence collected from infectious prions (reviewed in [28]). The neuroinvasion mechanisms associated with prions have been extensively described and largely depend of the administration route (reviewed in [28]). For example, while the intra-peritoneal administration of prions seems to require peripheral replication of the agent in lymphoid tissues (e.g., spleen) prior to neuroinvasion, prions administered by intra-gastric, intra-ocular or intra-neural (sciatic nerve) routes directly reach peripheral nerves and are transported to the brain either directly or after being replicated within the same pathways [28]. Although we acknowledge that peripheral to CNS transport of misfolded Aβ particles cannot be directly extrapolated to prion mechanisms (due to the ability of the latter to replicate in peripheral compartments), evidence on neural transport warrants further analyses. In this study, we administered Aβ seeds by intra-lingual and extra-nasal routes considering that the tissues involved are highly innervated. The olfactory sensory neurons present in the nasal cavity connect with the olfactory bulb in the brain, and the trigeminal nerve and autonomic nerves connect with the nasal epithelia [48]. The tongue is a highly innervated organ that received sensory and motor innervation from four cranial nerves [49]. Considering this, and our previous data demonstrating that eye exposure was the most efficient route of peripheral Aβ seeding when compared to three other routes [12], we evaluated the involvement of intra-lingual and extra-nasal administrations in the transport of Aβ seeding activity from the periphery to the brain.

Here, we demonstrated that intra-lingual administration of Aβ seeds increased overall brain amyloidosis in Tg2576 mice. These results were confirmed by two different but complementary techniques (immunohistochemistry and ELISA), providing rigor to this conclusion. This study was inspired by our previous report involving the administration of Aβ seeds through different routes of exposure and with dissimilar connectivity to the brain [12]. In that study, we observed that eye exposure was the most efficient route of seeding, only after intra-cerebral administrations. In line with those results, two additional routes with direct connectivity to the brain were explored. Unfortunately, the source of seeds used in the previously published experiments was not the same as the one used in the current study. Considering this, the seeding titers for both injectate are likely to be different, so direct comparisons between the data of both studies cannot be done.

Considering the high innervation and brain connectivity of the tongue, it is plausible that Aβ seeds administered in this organ directly reach the brain through retrograde transport. Although the overall deposition of Aβ in the brain did not change after the extra-nasal exposure of Aβ seeds, we observed an enrichment of the pathology in the olfactory bulb. Considering this result, and the above discussed connectivity between the nasal cavity and the brain, we hypothesize that misfolded Aβ can travel to the brain through retrograde axonal transport. Future studies will confirm or discard this experimental-based assumption. It is important to mention that mice treated with Aβ seeds by the extra-nasal route displayed variable brain amyloidosis. We believe that this variation is originated by the low-invasive nature of the procedure that results in a dissimilar amount of Aβ seeds incorporated by each animal. Specifically, we observed that some mice removed some volume of the injectate from the nose through exhalation. Along the same line, inhalation of the material (event that by obvious reason we were unable to measure) will also reduce the exposure of seeds to the nasal tissue. Along this line, a previous report show that chronic nasal administration of human Aβ synthetic peptide decreased brain amyloidosis in a mouse model of AD [47]. The authors hypothesized that the observed outcomes might be due to the activation of an immune response to Aβ after chronic nasal exposure, thus suggesting a promising immunological, therapeutic approach [47]. The discrepancies noted by this study and ours may be explained mostly by the single vs. chronic exposure of peptides. In addition, our study specifically included misfolded (seeding competent) Aβ aggregates. Moreover, both studies use different animal models (PDAPP [50] in the chronic exposure study). This is relevant, as both PDAPP and Tg2576 mice are likely develop different Aβ species that may alter the rate of amyloid pathology progression (reviewed in [51]).

It is important to consider that in addition to retrograde transport, peripherally administered prions can also reach the brain directly through the circulation [52,53] or other pathways. The nasal epithelia and tongue are rich in lymphatic tissue, and activated macrophages at that level could participate in the spread of Aβ seeds to the CNS. The formation of cerebral amyloid angiopathy after the intra-peritoneal and intra-venous exposure of Aβ seeds further suggest an active role of the circulation in brain uptake [12,54]. Intra-lingual and extra-nasal administrations are not likely to participate in iatrogenic transmissions as suggested for intra-muscular inoculations [22,46]. However, studying these routes provide us with valuable information on how misfolded Aβ particles are transported from the periphery to the brain. Whether this transport is due to either or both vascular and nervous components, and whether the transport mechanism is specific for the peripheral route being analyzed, is highly relevant. Research in this area will help us to understand the dynamics of misfolded Aβ in the context of AD with implications in disease mechanisms, diagnosis and risk assessment of potential iatrogenic transmissions.

## Figures and Tables

**Table 1:** Attack rate and brain amyloidosis induced by extra-nasal and intra-lingual administration of Aβ seeds in Tg2576 mice. Statistical analyses were performed using independent samples *t*-test (*p* < 0.05).

Group	n	Attack Rate	4G8 Burden Mean ± SD	Fold	*p* Value (Compared to Non-Treated)
Nasal	5	3/5	0.0977 ± 0.055	8.42	0.0769
Lingual	5	5/5	0.1157± 0.0745	9.95	0.0339
Untreated	4	-	0.0116 ± 0.016	1	-

## Data Availability

This study does not report any data.

## Supplementary Materials

The following supporting information can be downloaded at: https://www.mdpi.com/article/10.3390/cells11213442/s1, Figure S1: Aβ seeding activity of the inoculum in Tg2576 animals after intra-cerebral injection.; Figure S2: Aβ burden on the cortex or hippocampus of experimental animals.
