# Supplementary material for: Altering Brain Amyloidosis by Intra-Lingual and Extra-Nasal Exposure of Aβ Aggregates
Source: Cells. 2022 Oct 31;11(21):3442. doi: 10.3390/cells11213442 (PMC9654398; doi:10.3390/cells11213442)
Supplement: Supplementary file 1 [file cells-11-03442-s001.zip › cells-1933999-supplementary.pdf]

Supplemental Figure S1.

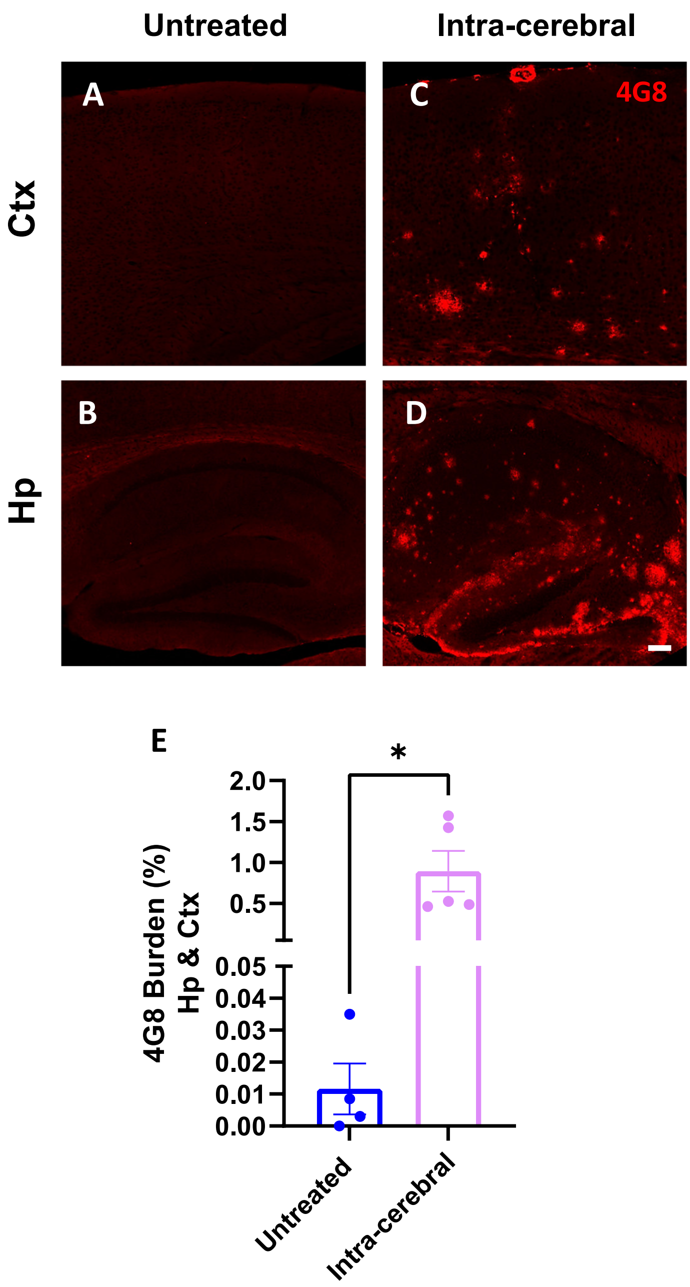

Supplemental Figure S1. Aβ seeding activity of the inoculum in Tg2576 animals after intra-cerebral injection. Representative pictures of 4G8 staining in cortex (A,C) and hippocampus (B,D) of

untreated, or intra-cerebrally injected Tg2576 mice. 4G8 burden (%) quantification in cortex and hippocampus (E) of untrated and intra-cerebrally challenged mice demonstrated the Aβ seeding activity of the. Values are expressed as mean ± SD. Statistical analyses were performed by independent samples t-test (*p<0.05). Scale bar represents 100 µm.

Supplemental Figure S2.

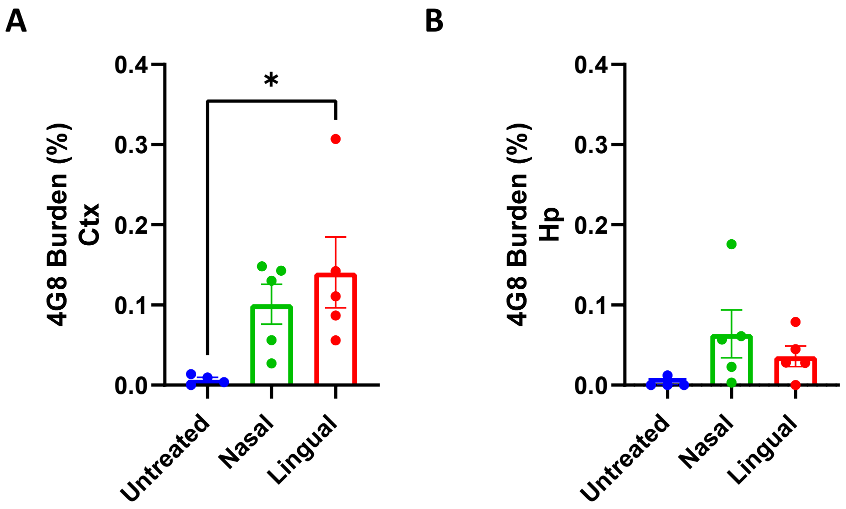

Supplemental Figure S2. Aβ burden in the cortex or hippocampus of experimental animals. 4G8 burden (%) quantification in cortex (A) and hippocampus (B) of 300-day-old Tg2576 mice challenged with Aβ seeds by the intra-lingual or extra-nasal routes. Representative pictures of these quantifications are displayed in Figure 2. One-way ANOVA and Tukey test (*p<0.05).
